# Protocol for rapid 5-plex 3D imaging and single-cell analysis of immune responses in whole murine lymph nodes

**DOI:** 10.1016/j.xpro.2025.104059

**Published:** 2025-09-01

**Authors:** Victoire Boulat, Alessandro Ciccarelli, Miu Shing Hung, Lingling Zhang, Anita Grigoriadis, Dinis Pedro Calado

**Affiliations:** 1 Cancer Bioinformatics, Faculty of Life Sciences and Medicine, King’s College London, London SE1 3SS, UK; 2 Immunity and Cancer Laboratory, The Francis Crick Institute, London NW1 1AT, UK; 3 Advanced Light Microscopy Facility, The Francis Crick Institute, London NW1 1AT, UK

**Keywords:** Cell Biology, Immunology, Microscopy

## Abstract

Lymph nodes orchestrate adaptive immune responses, with germinal centers enabling affinity maturation and plasma cell formation. Here, we present a protocol for rapid, high-resolution, multicolor 3D imaging of whole immunized mouse lymph nodes. We describe steps for immunization, lymph node harvesting, fixation, permeabilization, staining, and clearing. We cover image acquisition using a light sheet fluorescence microscope and analysis using the Imaris software. This protocol allows the quantification of germinal center B cells, plasma cells, and follicular T cells at single-cell resolution.

## Highlights

• Steps for rapid 3D multicolor light sheet imaging of whole immunized lymph nodes
• Instructions for optimized antibody-based protocol compatible with diverse genotypes
• Procedure for Imaris-based image analysis enabling segmentation of key immune subsets
• Guidance on quantification of germinal centers, follicular T cells, and plasma cells

## Abstract (editor highlights)

Publisher’s note: Undertaking any experimental protocol requires adherence to local institutional guidelines for laboratory safety and ethics.

## Graphical abstract

**Figure:**
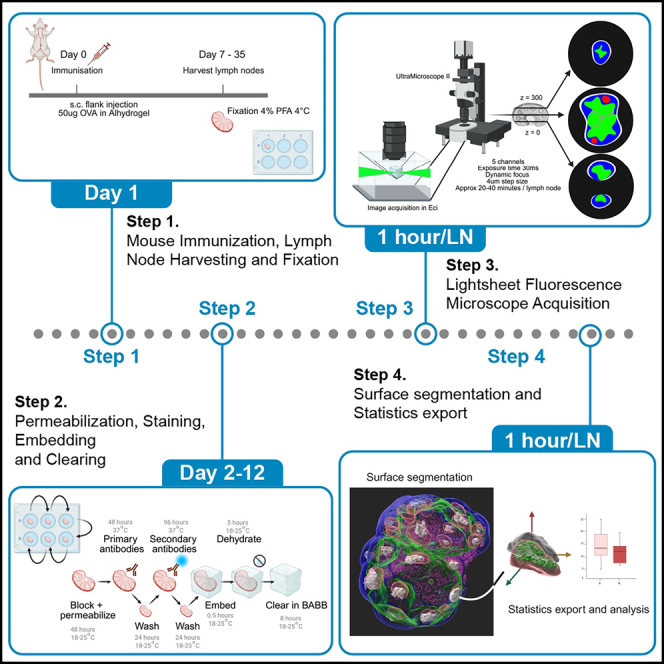

## Before you begin

Lymph nodes are highly organized secondary lymphoid organs that coordinate immune responses to pathogens, vaccines, and tumors.^1^^,^^2^^,^^3^ Within lymph nodes, spatial compartmentalization ensures efficient interactions between follicular dendritic cells (FDCs), B cells, T cells, and stromal cells, creating specialized microenvironments essential for immune activation and regulation.

A hallmark of adaptive immunity is the germinal center reaction, initiated by antigen-stimulated B cells in lymph node follicles.^3^ Germinal centers support affinity maturation, and differentiation into memory B cells and plasma cells. These dynamic structures are divided into light and dark zones, each characterized by distinct cellular populations and molecular programs. Light zones contain FDCs and T follicular helper cells (Tfh), which guide B cell selection and survival.^3^

Conventional 2D immunofluorescence often fails to resolve the full architecture of germinal centers, especially the spatial relationship between zones and associated immune subsets. 3D volumetric imaging is therefore essential to accurately capture germinal center dynamics, particularly in response to immunization or disease. Indeed, germinal center architecture, including the spatial distribution of important cell types may be disrupted in the context of cancer,^4^ autoimmunity,^5^ aging^6^ or infection^7^ and this is often not accurately represented in 2D.

This protocol provides a reproducible pipeline for rapid, multicolor single-cell resolution imaging of whole murine lymph nodes using light sheet fluorescence microscopy. It enables segmentation of germinal center B cells, Tfh cells, plasma cells, and key stromal structures such as lymphatics, vasculature, and the subcapsular sinus. Importantly, it is compatible with multiple genotypes and does not rely on transgenic markers, making it broadly applicable for studies of infection, vaccination, autoimmunity, and cancer.

### Innovation

While the molecular mechanisms of B cell maturation have been extensively characterized in the past decades, spatial information about this process remains limited and largely derived from 2D imaging. Given the germinal center’s spherical nature and distinct functional zones, we believe studying it in 3D will provide significant advances to the field. This protocol provides a rapid, high-resolution, multicolor approach for whole-mount 3D imaging of immunized mouse lymph nodes using light sheet fluorescence microscopy.

The use of a light sheet fluorescence microscope greatly reduces imaging time and data size while conserving single cell resolution. Indeed, imaging a single whole lymph node via confocal microscopy may take up to 12 hours^8^ with varying success depending on imaging depth as compared to 30 minutes via light sheet.

Unlike existing light sheet fluorescence microscopy workflows which rely on genetically modified mice or adoptive transfers to visualize similar lymph node structures and cell populations, this protocol enables the simultaneous detection of five intracellular and extracellular markers using antibody staining. This flexibility broadens its application to diverse mouse genotypes, such as those used in cancer or autoimmunity models, without requiring complex breeding strategies.

In addition, we provide a beginner-friendly and highly reproducible image analysis pipeline in Imaris, which includes machine learning segmentation. To overcome the limitations of statistics export in Imaris, we introduce a custom R workflow which batch concatenates statistics across lymph nodes. Together, this protocol offers a flexible, high-throughput workflow for detailed 3D immunophenotyping of mouse lymph nodes.

### Institutional permissions (if applicable)

All mice used in this work were housed at The Francis Crick Institute. All animal experiments described in this protocol were approved by the Francis Crick Institute Biological Resources Facility strategic oversight committee (which incorporates the Animal Welfare and Ethical Review Body) and by the Home Office, UK. All mouse experiments must be conducted with the approval of the animal care committee at your research institution. Adhere to local guidelines for safe handling and disposal of dangerous materials. When applicable, a risk assessment should be completed for the use of specific materials.

### Mouse immunization, lymph node harvest, and fixation

**Timing: 0–1 day after harvesting lymph nodes**

**Figure 1:**
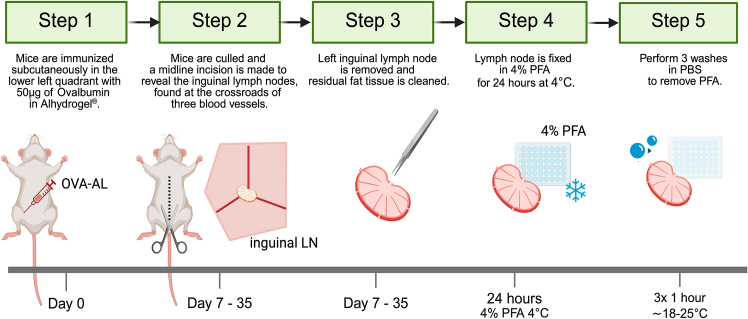
Workflow for immunization with Ovalbumin in Alhydrogel, harvesting and fixation of the inguinal lymph node

Here, we describe the protocol for the immunization, harvesting and fixation of the immunized inguinal lymph nodes (Figure 1).

1. Immunize adult mice (8–12 weeks old) subcutaneously in the lower left quadrant with 50 μg of Ovalbumin in Alhydrogel, as outlined in the materials and equipment section.
  a. Load a syringe with the prepared immunization solution and attach a 25-gauge needle.
  b. Restrain the mouse using the scruffing technique: hold the tail, allow it to grip a hard surface, then grasp the scruff of the neck securely while tucking the tail behind the pinkie finger.
  c. Rotate the mouse so its abdomen is facing upwards. Mentally divide the abdomen into 4 quadrants.
  d. Remove the needle cap and, maintaining a shallow angle, insert the needle into the lower left quadrant. Inject 100 μl of the solution subcutaneously.

***Note:*** A visible bleb should form under the skin at the injection site. If this is not the case, the injection may have been intraperitoneal which would result in inefficient targeting of the inguinal lymph node.

2. Cull and dissect mice 7–35 days post-immunization.
  a. Place mice on a dissection board and sterilize the skin with 70% ethanol.
  b. Using surgical scissors, make an incision through the first layer of skin from the tail to the ribcage, taking care not to cut through the peritoneum.
  c. Gently lift the skin on either side to expose the underlying tissue.
3. Using surgical forceps, locate and remove the left inguinal lymph node.

***Note:*** The inguinal lymph node is positioned at the junction of three blood vessels and appears slightly different in color from the surrounding fat pad. A more detailed protocol for harvesting the inguinal lymph node can be found elsewhere.^9^^,^^10^

4. Carefully remove any residual fat tissue using forceps. Place the cleaned lymph nodes into ice-cold PBS in a 48-well plate for further processing.

***Note:*** The inguinal lymph node from the other side can be dissected and used as a non-immunized control. Biological replicates can be placed into the same well to minimize reagent volume.

5. Transfer lymph node to a new well of the 48-well plate containing 500 μl Fixation buffer (4% PFA in PBS). Incubate on a shaker at 4°C for 12–16 hours.

**CRITICAL:** Over-fixation and excessive handling can cause tissue damage. Troubleshooting 1.

6. Transfer lymph node out of Fixative solution and transfer to a new well containing 500 μL PBS. Incubate on a shaker at 4°C for 1 hour. Repeat twice for a total of 3 washes.

## Key resources table

**Table:** 

REAGENT or RESOURCE	SOURCE	IDENTIFIER
**Antibodies**		

Alexa Fluor 488 anti-mouse Bcl6 (clone K112-91) (dilution: 1:25)	BD Biosciences	Cat#561524
Alexa Fluor 594 anti-mouse CD3 (17A2) (dilution: 1:50)	BioLegend	Cat#100240
Alexa Fluor 647 anti-mouse CD138 (281-2) (dilution: 1:50)	BioLegend	Cat#142526
Alexa Fluor 700 anti-mouse IgD (clone 11-26c.2a) (dilution: 1:100)	BioLegend	Cat#405730
Anti-mouse CD35+CD21 rabbit antibody (clone 7G6) (dilution: 1:25)	Abcam	Cat#ab288422
Goat anti-rabbit IgG (H+L) highly cross-adsorbed secondary antibody, Alexa Fluor 790 (dilution: 1:600)	Thermo Fisher Scientific	Cat#A11369
Alexa Fluor 488 anti-mouse Lyve1 (clone ALY7) (dilution: 1:100)	Thermo Fisher Scientific	Cat#53-0443-82
Alexa Fluor 594 anti-mouse CD169 (clone 3D6.112) (dilution: 1:100)	BioLegend	Cat#142416
Alexa Fluor 647 anti-mouse CD31 (clone 390) (dilution: 1:100)	BioLegend	Cat#102416
Alexa Fluor 594 anti-mouse B220 (clone RA3-6B2) (dilution: 1:100)	BioLegend	Cat#103254
Alexa Fluor 647 anti-mouse CD4 (RM4-5) (dilution: 1:50)	BioLegend	Cat#100530
Alexa Fluor 647 anti-mouse CD8 (53-6.7) (dilution: 1:50)	BioLegend	Cat#100758
Alexa Fluor 647 anti-mouse Bcl6 (clone K112-91) (dilution: 1:25)	BD Biosciences	Cat#561525
Mouse IgG1 isotype control (MOPC-21), Alexa Fluor 488 (dilution: 1:25)	Thermo Fisher Scientific	Cat#MA5-18167
Alexa Fluor 594 rat IgG2b, κ isotype Ctrl antibody (dilution: 1:50)	BioLegend	Cat#400661
Alexa Fluor 647 rat IgG2a, κ isotype Ctrl antibody (dilution: 1:50)	BioLegend	Cat#400526

**Biological samples**		

BALB/c mice (lymph nodes) (wild-type, male and female, 8–12 weeks)	The Francis Crick Institute	Strain #:000651 from Jackson Laboratories

**Chemicals, peptides, and recombinant proteins**		

Ovalbumin	Sigma-Aldrich	Cat#A7641-50MG
Saline	Hameln	Cat#SC010
Alhydrogel adjuvant 2%	InvivoGen	Cat#vac-alu-50
Paraformaldehyde (PFA)	Thermo Fisher Scientific	Cat#043368.9M
PBS	Gibco	Cat#10010023
Ce3D permeabilization/blocking buffer	BioLegend	Cat#427706
Ce3D wash buffer	BioLegend	Cat#427709
Ce3D antibody diluent buffer	BioLegend	Cat#427708
UltraPure low melting point agarose	Invitrogen	Cat#16520050
Methanol	Sigma-Aldrich	Cat#34860-1L-R
Benzyl alcohol (BA)	Sigma-Aldrich	Cat#24122-M
Benzyl benzoate (BB)	Thermo Fisher Scientific	Cat#105860010
Ethyl cinnamate (ECi)	Sigma-Aldrich	Cat#112372-100G
Triton X-100	Thermo Fisher Scientific	Cat#9002-93-1
Normal mouse serum	Invitrogen	Cat#11869220
Thioglycerol	Sigma-Aldrich	Cat#M1753-100ML

**Experimental models: Organisms/strains**		

BALB/c mice (wild-type, male and female, 8–12 weeks)	The Francis Crick Institute	Strain #:000651 from Jackson Laboratories

**Software and algorithms**		

ImSpector software	Abberior Instruments GmbH	Imspector Image Acquisition & Analysis Software
Imaris 10.1 or higher	Oxford Instruments	https://imaris.oxinst.com
Imaris File Converter	Oxford Instruments	https://imaris.oxinst.com
R (version 3.6+)	Open-source software	https://cran.r-project.org
GitHub README file for custom R workflow	GitHub	https://github.com/FrancisCrickInstitute/Imaris-3D-LN-stats and https://doi.org/10.5281/zenodo.16789543

**Other**		

Concentric disposable syringe 1 mL	Terumo	Cat#SYR6200
Microlance 3 25G 1″ needles	BD Biosciences	Cat#12389169
48-well flat bottom cell culture plate	Falcon	Cat#353078
15 mL conical centrifuge tubes	Falcon	Cat#10136120
Peel-A-Way embedding molds	Sigma-Aldrich	Cat#E6032-1CS
LevGo smartSlicer safety razor blades	Fisher Scientific	Cat#15326558
Miltenyi-LaVision BioTec UltraMicroscope II, microscope body (Olympus MVX-10), detection objective (MVPLAPO 2X NA 0.5), dipping cap (WD > 5.7 mm, magnification 1.125x), magnification changer (steps from 0.63x to 6.3x), detector (pco.edge 5.5M pixel camera with 2,560 × 2,160 px where px size = 6.5 μm)	Miltenyi	N/A
Cuvette and sample holder set for Super Plan Module	Miltenyi	Cat#130-133-604
Computer for image acquisition (i7 Intel Core, 32 GB RAP, 256 SATA SSD, 2 TB HD hard drive)	N/A	N/A
Computer for image analysis (Windows 10 work station with a high-performance CPU (3.3 GHz with 12 to 16 cores), ample RAM (64–128 GB), a dedicated GPU with sufficient VRAM)	N/A	N/A

## Materials and equipment

### Ovalbumin in Alhydrogel

Prepare in a sterile environment. Dissolve Ovalbumin in saline or PBS to a final concentration of 10 mg/mL. Sterile filter the solution using a 0.22 μm filter. Dilute the Ovalbumin stock solution 1:10 in saline or PBS to obtain a 1 mg/mL solution. Mix this diluted solution 1:1 with Alhydrogel. Ensure thorough mixing to achieve a homogeneous suspension. Make fresh and store the prepared Ovalbumin-Alhydrogel mixture at 4°C until injection.

### Fixation buffer

4% PFA in PBS. Make fresh and store in the dark at 4°C for up to two weeks.

**Permeabilization buffer** is Ce3D Permeabilization/Blocking Buffer. Permeabilization buffer is stored at 4°C.

### Antibody cocktail 1

**Table:** Enough for 1 well of a 48-well plate which can hold up to 4 lymph nodes

Reagent	Final concentration	Amount
Anti-mouse CD35+CD21 rabbit antibody (clone 7G6)	1:25	16 μL
Ce3D Antibody Diluent Buffer	N/A	384 μL
**Total**	**N/A**	**400 μL**

Antibody cocktail must be made fresh and stored at 4°C on the day each time.

### Antibody cocktail 2

**Table:** Enough for 1 well of a 48-well plate which can hold up to 4 lymph nodes

Reagent	Final concentration	Amount
Alexa Fluor 488 anti-mouse Bcl6 (clone K112-91)	1:25	16 μL
Alexa Fluor 594 anti-mouse CD3 (17A2)	1:50	8 μL
Alexa Fluor 647 anti-mouse CD138 (281-2)	1:50	8 μL
Alexa Fluor 700 anti-mouse IgD (clone 11-26c.2a)	1:100	4 μL
Goat anti-Rabbit IgG (H+L) Highly Cross-Adsorbed Secondary Antibody, Alexa Fluor 790	1:600	0.65 μL
Ce3D Antibody Diluent Buffer	N/A	363.35 μL
**Total**	**N/A**	**400 μL**

Antibody cocktail must be made fresh and stored at 4°C on the day each time.

**Table:** Antibody controls

Antibody control	Type of control used	Antibody isotype	Primary antibody
Mouse IgG1 Isotype Control (MOPC-21), Alexa Fluor 488	Isotype control	Mouse IgG1	Alexa Fluor 488 anti-mouse Bcl6 (clone K112-91)
Alexa Fluor 594 Rat IgG2b, κ Isotype Ctrl Antibody	Isotype control	Rat IgG2b	Alexa Fluor 594 anti-mouse CD3 (17A2)
Alexa Fluor 647 Rat IgG2a, κ Isotype Ctrl Antibody	Isotype control	Rat IgG2a	Alexa Fluor 647 anti-mouse CD138 (281-2)
Alexa Fluor 647 Rat IgG2a, κ Isotype Ctrl Antibody	Isotype control	Rat IgG2a	Alexa Fluor 700 anti-mouse IgD (clone 11-26c.2a)
Goat anti-Rabbit IgG (H+L) Highly Cross-Adsorbed Secondary Antibody, Alexa Fluor 790	Secondary antibody alone	N/A	Anti-mouse CD35+CD21 rabbit antibody (clone 7G6)

Wash buffer is Ce3D Wash Buffer. Wash buffer is stored at 4°C.

### 2% low melting point agarose

Prepare a 2% Low Melting Point Agarose solution by dissolving 2 g of UltraPure Low Melting Point Agarose in 100 mL of deionized water in a 500 mL Duran bottle. Mix thoroughly and place the bottle in the microwave with the cap loosely positioned on top. Heat at maximum power in 20-second increments, checking after each cycle, until the agarose is fully dissolved. The solution should be bubbling and hot. To cool the agarose safely, hold the Duran bottle under a stream of cold tap water, ensuring the water runs over the bottom of the bottle while avoiding contamination. Continue cooling until the bottle is no longer hot to the touch, approximately 37°C.

### Methanol dilutions

Prepare methanol dilutions using deionized H_2_O. These dilutions can be stored at 18°C–25°C in a flammables cabinet until needed.

### Clearing solution

Prepare BABB by mixing benzyl alcohol (BA) and benzyl benzoate (BB) in a 1:2 ratio.^11^ For example, to prepare 240 mL of BABB, mix 80 mL of BA with 160 mL of BB. The mixture can be stored at 18°C–25°C in a flammables cabinet for up to two months.

**CRITICAL:** Benzyl alcohol, benzyl benzoate, and methanol are hazardous chemicals. Always handle them inside a fume hood to ensure safety. Benzyl alcohol, benzyl benzoate, and methanol can melt some polystyrene-based plastics so their use with these materials is to be avoided. Glass or polypropylene containers should be used instead.

**Imaging solution** is Ethyl Cinnamate (ECi) which can be stored at 18°C–25°C for extended periods without degradation.

**CRITICAL:** despite not being a hazardous material, ECi can melt some polystyrene-based plastics so its use with these materials is to be avoided. Glass or polypropylene containers should be used instead.

### Alternative buffers

• Permeabilization/Blocking Buffer & Antibody Diluent Buffer Alternative: Supplement 1× PBS with 0.3% Triton X-100, 1% normal mouse serum, and 1% bovine serum albumin. Store at 4°C.
• Wash Buffer Alternative: Supplement 1× PBS with 0.5% thioglycerol and 0.3% Triton X-100. Store at 4°C.

### Light sheet fluorescence microscope specifications and setup

Here, we used the LaVision Biotec UltraMicroscope II Light sheet Fluorescence Microscope. LaVision Biotec was acquired by Miltenyi in 2018 who now sell the UltraMicroscope II. A comprehensive guide to the microscope compiled by an enthusiastic user was published in 2018 and may be a useful resource for new users.^12^

The UltraMicroscope II specifications and set-up used in this protocol is as follows.

• Detection objective: MVPLAPO 2x NA 0.5.
• Dipping cap: WD>5.7 mm, magnification 1.125x.
• Magnification changer: Steps from 0.63x to 6.3x.
• Detector: PCO Edge 5.5M pixel camera with 2560x2160 pixels where pixel size = 5.5 μm).
• Illumination lasers: 488 nm, 561 nm, 638 nm, 705 nm, 785 nm.
• Detection fluorescence filters: GFP 525/50, Tomato 620/60, FarRed 680/30, F-FarRed0 740/40, E-FarRed2 835/70.
• Light-sheet illumination: 3 left-side light sheets and beam thickness set to 4 μm.

**Table:** The laser-filter set-up required for this protocol is as follows

Fluorophore	Excitation laser	Emission filter
AF488	488	525/50
AF594	561	620/60
AF647	640	680/30
AF700	705	740/40
AF790	785	835/70

***Note:*** The system must be pre-aligned for imaging in ECi (RI 1.558) and the setting for this liquid has to be selected in the software menu; however, for more precise Light sheet alignment, we recommend at the beginning of each session to align the Light sheet on the sample (see Step 20).

## Step-by-step method details

### Permeabilization, staining, embedding, and clearing

**Timing: 2–12 days after harvesting lymph nodes**

**Figure 2:**
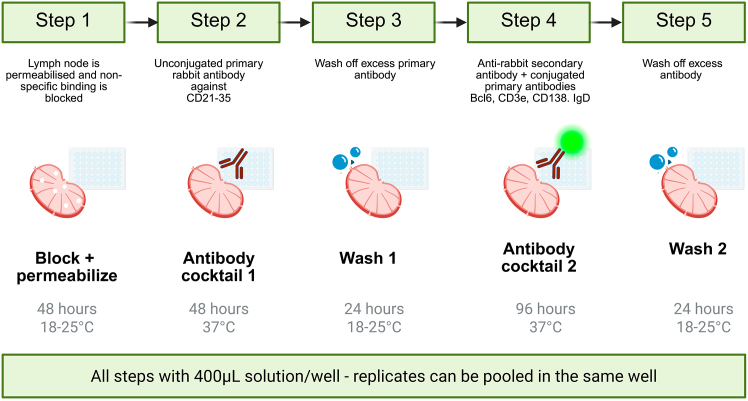
Workflow for blocking, permeabilization, and antibody staining of lymph nodes in 48-well plates

**Figure 3:**
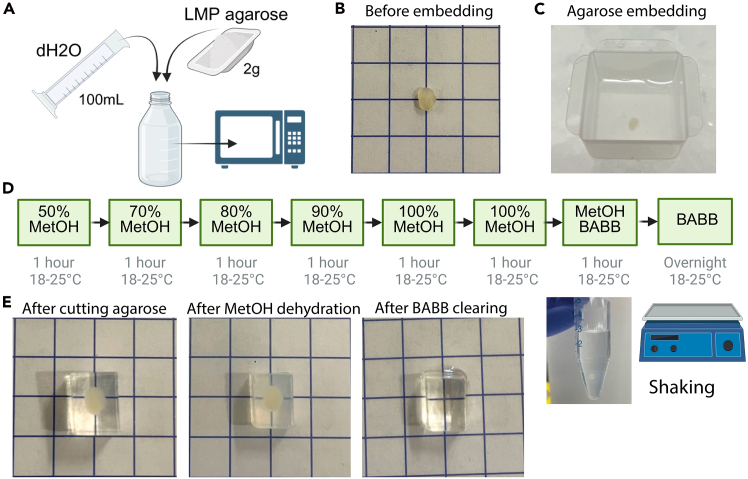
Embedding, dehydration, and clearing of lymph nodes for light sheet imaging (A) Schematic of the making of 2% Low Melting Point agarose. (B) Photograph of an inguinal lymph node before agarose embedding. (C) Photograph of an inguinal lymph node in agarose in an embedding mold on ice. (D) Schematic representation of the dehydration and clearing steps including a photograph of an agarose-embedded lymph node immersed in 50% Methanol in a 15 mL Falcon tube. (E) Photographs of the agarose-embedded lymph node after demolding, after dehydration and after 16-hour BABB incubation. The agarose may become partially opaque after dehydration and then both the agarose and the lymph node become completely transparent after clearing. Paper grid squares: 0.5 cm x 0.5 cm.

Here we describe the protocol for permeabilization, staining (Figure 2), embedding, dehydration and clearing (Figure 3) of the lymph nodes.

1. Transfer lymph node to a new well containing 500 μL Permeabilization/Blocking Buffer. Incubate at 18°C–25°C on a shaker for 48 hours.

***Note:*** A dedicated Fc block step is not included, as we did not observe non-specific binding attributable to Fc receptor interactions. In particular, staining of FDCs was only detected in the expected channel when using the anti-CD21/35 antibody, and not in isotype or secondary-only controls (Figure S1). However, if users encounter background staining on FDCs or Fc receptor-expressing cells, adding Fc block (anti-mouse CD16/CD32) to the blocking buffer may be beneficial.

2. Transfer lymph node to a new well containing 400 μL Antibody Cocktail 1 (which contains the rabbit primary antibody against CD21/35). Incubate on a shaker at 37°C for 48 hours at 100–150 rpm.

**CRITICAL:** To avoid antibody aggregates, centrifuge all antibody cocktails once made for 3 minutes at 10,000 x g. Troubleshooting 2.

**CRITICAL:** At all steps, but especially at 37°C, the plate must be covered with parafilm to avoid evaporation.

3. Transfer lymph node to a new well containing 500 μL Wash Buffer. Incubate on a shaker at 18°C–25°C for 24 hours, changing the wash buffer twice (8 hour per wash approximately).
4. Transfer lymph node to a new well containing 400 μL Antibody Cocktail 2 (which contains the fluorescent antibodies against Bcl6, CD3, IgD, CD138 and the secondary anti-rabbit AF790 antibody). Incubate on a shaker at 37°C for 96 hours.

***Note:*** Incubation times and antibody concentrations can be modulated if the final acquisition reveals weak signal or lack of staining. Troubleshooting 3.

**CRITICAL:** Fluorescently labeled antibodies are light-sensitive and can undergo photobleaching, leading to reduced signal intensity. From this step onwards, all steps must be performed while minimizing exposure to light. To do so, cover the well plate with foil.

5. Transfer lymph node to a new well containing 500 μL Wash Buffer. Incubate on a shaker at 18°C–25°C for 24 hours, changing the wash buffer twice (8 hour per wash approximately).

**Pause point:** Samples can be stored in Wash Buffer at 4°C for up to one week.

6. Make the 2% Low Melting Point agarose solution with the use of a microwave (Figure 3A). Cool the solution to approximately 37°C. Use a Pasteur pipette to cover the bottom of a Peel-Away embedding mold with the solution.
7. Dry the lymph node on a KimWipe and place in the cube. Place on a flat surface in an ice bucket (Figures 3B and 3C).

**CRITICAL:** Work quickly so the agarose does not solidify before the lymph node is fully submerged.

8. Trim the agarose-embedded lymph node.
  a. Cut the embedding cube open to free the agarose block which contains the lymph node.
  b. Using a razor blade, cut the agarose around the lymph node to form a small cube containing only the lymph node.
    ***Note:*** Ensure that the lymph node is oriented so that its longest plane is parallel to the sides of the agarose cube. It should not be at an angle within the block.
  c. Trim the cube as in Figure 3E.
9. Place agarose cube in a 15-ml polypropylene Falcon tube.

***Alternatives:*** The solutions used for dehydration and clearing damage polystyrene-based plastics so glass tubes can also be used for these steps.

10. Incubate agarose block in the following solutions for 1 hour each at RT on a shaker set at 100 rpm (Figures 3D and 3E).^11^
  a. 50% MetOH.
  b. 70% MetOH.
  c. 80% MetOH.
  d. 90% MetOH.
  e. 100% MetOH.
  f. 100% MetOH.
  g. 50% MetOH – 50% Clearing solution.
11. Transfer agarose block to Clearing solution (BABB).^11^ Incubate for 12–16 hours on a shaker at 18°C–25°C (Figures 3D and 3E).

***Note:*** Two to three hours in clearing solution may be sufficient to achieve full clearing of the agarose-embedded lymph node. Matching incubation times across experiments is crucial to minimize differential shrinkage.

***Note:*** While the blocking, staining, and washing steps in this protocol utilize Ce3D reagents, the clearing solution used is not the Ce3D clearing solution due to its incompatibility with the light sheet fluorescence microscope setup. The UltraMicroscope II requires the sample to be immersed in an imaging chamber containing approximately 130 mL of imaging solution, with a refractive index that matches the clearing solution. While Ethyl cinnamate is a good match for BABB, no suitable match was found for the Ce3D clearing solution. Additionally, the Ce3D clearing solution is highly viscous, making it difficult to handle.

12. Transfer agarose block to Imaging solution for 5 minutes and then to clean Imaging solution for an hour on a shaker at 18°C–25°C.

***Note:*** Methanol and Clearing solution are harmful and toxic. A chemical fume hood should be used for all steps where they are used.

***Note:*** During the dehydration, the agarose block might become partially opaque.

**Pause point:** Fluorescent signal is stable for multiple months post-clearing.

### Light sheet microscope acquisition

**Timing: 20–40 min per lymph node**

**Figure 4:**
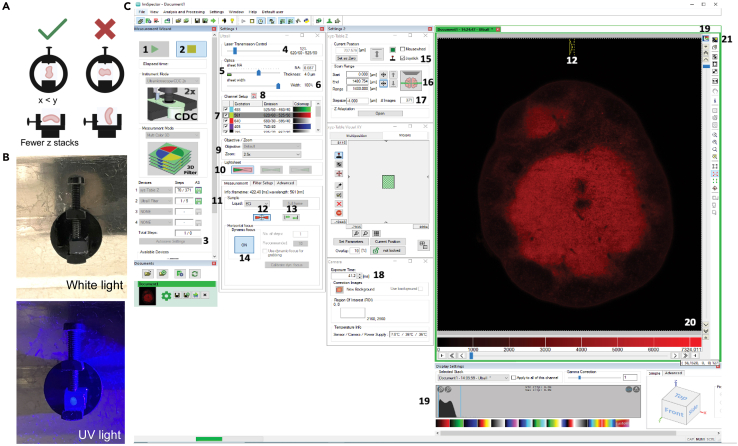
Sample mounting and imaging settings for light sheet fluorescence acquisition (A) Diagram showing correct sample orientation on sample holder. (B) Photographs of agarose-embedded lymph node in the sample holder under white and UV light. (C) ImSpector software screengrab showing setting used to image whole lymph nodes. 1: Start acquisition, 2: Live preview, 3: Save settings , 4: Laser power, increases values in histogram (19), 5: Light sheet thickness, decreases width of butterfly (12), 6: Width, always 100%, 7: Channel selection, highlighted channel is live, ticked channels will be acquired , 8: Chromatic correction tool, set focus with focus knob for AF488 and adjust with this for all the other channels, 9: Zoom, set to actual, 10: Light sheet selection, 11: Liquid is ECi, 12: Use the butterfly to check the alignment of the light sheet (the column under the butterfly should be most in focus), 13: If using dynamic focus, set the range here, 14: Use this button to toggle dynamic focus on and off and set the number of steps desired (follow recommended), 15: Use the z knob on the joystick to set the first stack where the lymph node is visible as 0, 16: Use the z knob on the joystick to set the start and end of the acquisition , 17: Set the z step thickness (4 μm is usually sufficient), 18: Set exposure time as low as possible, 19 + 20: Use the histogram to adjust the display and choose laser power based on the intensity values (positive signal should be between 1000 and 10000 a.u.), 21: Use the auto adjust contrast button to optimize the image display.

Here, we describe the protocol for the image acquisition of the stained whole immunized lymph nodes using the UltraMicroscope II light sheet fluorescence microscope, including microscope and sample set-up and optimized illumination and acquisition settings (Figure 4).

13. Place dipping lens on microscope objective.
14. Fill imaging chamber with Imaging solution.
15. Place sample in the imaging chamber.
  a. Using the diagram provided in Figure 4A, place the agarose block containing the cleared lymph node into the sample holder. Use a UV torch to illuminate the transparent lymph node and orient it correctly (Figure 4B). Secure the agarose block with a small screw.
  b. Secure the sample holder onto the immersion holder with a screw and place into the imaging chamber.
16. On the ImSpector software, set channel intensity to 2% (“4” in Figure 4C) and capture (“2” in Figure 4C). Use joystick dial to place sample in the light sheet’s path.

**CRITICAL:** While setting up the image acquisition, it is important to keep moving the sample in Z to avoid over-illuminating the same Z plane and bleaching the sample. AF700 is especially sensitive to photobleaching.

17. Lower the dipping lens until it is immersed in the Imaging solution. Keep an eye on the viewer tab of the software and adjust display settings until you see the lymph node (“21” in Figure 4C).
18. Center the lymph node in x and y.
19. Increase objective zoom until the lymph node fills the entire field of view of the objective. Use the joystick dial to verify that the whole sample fits in the frame throughout z (from top to bottom).
20. Adjust the value of the zoom on the software as well (“9” in Figure 4C).

***Note:*** While tiling is possible, due to its time-consuming nature, it is not recommended. The highest magnification which fits the whole sample should be chosen. Usually, the whole lymph node fits using the 3.2x zoom.

21. Set the sample liquid to ECi (“11” in Figure 4C) and use the Advanced tab to align the light sheet.
  a. Move the icon representing the waist of the light sheet (named for simplicity in our microscopy facility “butterfly”) over an area of the sample where there is good and homogenous signal.
  b. Move left to right the sheet motor calibration tool (under the Advanced tab in “11” in Figure 4C) and stop when the column showing the brightest signal/highest contrast is beneath the butterfly.

***Note:*** The butterfly icon represents both the waist position and thickness of the light sheets used.

**CRITICAL:** Despite already selecting the Liquid in the software, it is recommended to check and possibly re-align the light sheet on the sample every time the microscope is used. It does not need to be re-aligned for each sample acquired that day.

22. Tick appropriate channels (appropriate for AF488, AF594, AF647, AF700 and AF790, see Light sheet fluorescence microscope set-up) (“7” in Figure 4C).

***Note:*** The highlighted channel is the one which is currently being viewed while the ticked channels will be acquired during an acquisition.

23. Choose light sheet setting. We use the left side illumination and all three light sheets (“10” in Figure 4C). Set the beam thickness to 4 μm (“5” in Figure 4C).

***Note:*** We use single-sided illumination because the lymph nodes are narrow and well cleared, allowing light to pass through easily and both sides to remain in focus. For wider or poorly cleared samples, image quality may degrade on the far side. In this case, dual-sided illumination can improve results, at the cost of longer imaging time and increased risk of photobleaching. For single-sided illumination, the side from which the light sheet is cast is not critical but, must be kept consistent in a single imaging batch.

***Note:*** We use all three light sheets to reduce striping artefacts caused by perturbations in light propagation such as bubbles or debris in the imaging solution.

***Note:*** The beam thickness is set to 4 μm to provide a balance between axial resolution and signal-to-noise ratio, allowing for clear visualization of substructures without excessive photobleaching or background.

24. Set up dynamic focus.
  a. Define the area to image with the dynamic focus by positioning the horizontal focus boundaries on the left and right side of the sample in the Z plane where it is widest (“13” in Figure 4C).
  b. Set number of dynamic focus steps to the recommended number for the AF647 channel (“14” in Figure 4C). In our settings we use 8 dynamic focus steps.
  c. Set as dynamic focus merging algorithm “blend”.
25. Set exposure time to 30 ms (“18” in Figure 4C). The software will automatically slightly adjust exposure time.

***Note:*** To speed up acquisition, it is preferable to use short exposure times and high laser powers. There is only one exposure time for all channels.

26. Set channel focus. For this step, to avoid photobleaching, keep laser brightness low (approximately 5%). Troubleshooting 4.
  a. Start with Bcl6-AF488. Use the focus knob and the butterfly (“12” in Figure 4C) to set focus where individual cells are clearest below the focus butterfly.
    **CRITICAL:** Once set for the AF488 channel, do not adjust focus at the microscope knob.
  b. Proceed through the channels in order of increasing wavelength (CD3-AF594, CD138-AF647, IgD-AF700, CD21-35-AF790). Adjust the focus for each by using the chromatic correction tool (“8” in Figure 4C).
27. Set the Z step size to 4 μm, which despite not optimal, is sufficient for extracting from the images the information we required (“17” in Figure 4C).
28. Define the z-range by using the joystick dial to scroll to the first plane where the lymph node becomes visible and set this as the first z-plane. Continue scrolling to the last plane where the lymph node remains visible and set this as the last z-plane (“16” in Figure 3C).

***Note:*** An entire lymph node should fit into approximately 400 slices.

**CRITICAL:** For this step, to avoid photobleaching, keep laser power low (approximately 5%).

29. Set channel laser power (“4” in Figure 4C). Do this step last to avoid photobleaching the sample. Use the histogram (“19+20” in Figure 4C) to determine the appropriate power.

***Note:*** As a general rule, for each channel select the lowest laser power necessary to have the objects of interest (e.g., cell bodies) with an intensity between 1000 and 10000 a.u. The laser power required for the CD3 channel (AF594) will be much lower than the others. As a reference, we regularly use these laser powers: Bcl6-AF488: 75%, CD3-AF594: 15%, CD138-AF647: 40%, IgD-AF700: 45%, CD21-35-AF790:70%.

***Note:*** The final voxel size was 0.94 x 0.94 x 4 μm, lymph node volume was approximately 4 mm^3^.

**CRITICAL:** Use the same laser power for each channel across samples to enable comparison of channel intensities.

30. Set save settings (“3” in Figure 4C) and start acquisition (“1” in Figure 4C). One lymph node should be acquired in 20–40 minutes.

### Image analysis: Surface segmentation

**Timing: 1 h per lymph node**

Here, we describe the image analysis pipeline using Imaris for surface segmentation of the following structures and cell populations: whole lymph node, T cell zone, naïve B cells, FDCs, germinal centers, plasma cells and follicular T cells (Figures 5, 6, 7, 8, 9, 10, 11, 12, and 13).

**Figure 5:**
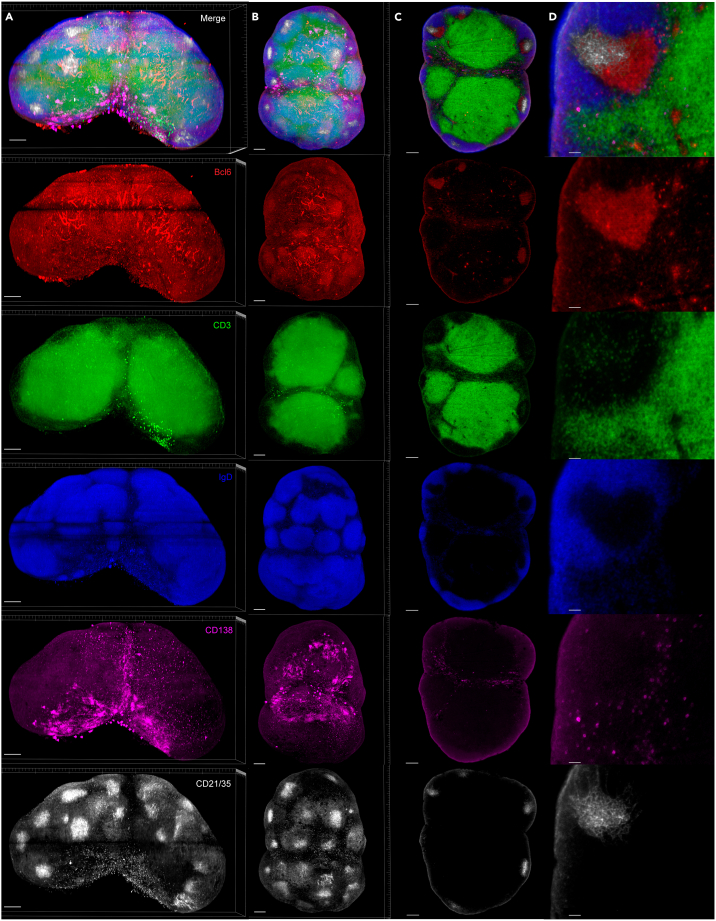
3D and 2D visualization of multicolor stained lymph nodes showing germinal center structures Representative 3D (A and B), 2D (C) and cropped images (D) of the merged and individual channels. The first two columns (A and B) are 3D renderings shown as maximum intensity projections). In column A, the bottom axis represents the y-axis, while the side axis represents the z-axis. In column B, the bottom axis represents the y-axis, and the side axis represents the x-axis. The last two columns (C and D) are 2D images of single planes. The CD3 and the CD138 channels enable identification of single cells when distribution is sparse. In the first column, the dark horizontal line is caused by excess illumination of a single plane. How to avoid this is covered in the troubleshooting problem 4. A, B and C column scale bar = 200 μm, 4^th^ column scale bar = 40 μm.

31. Use Imaris File Converter to convert the stack of TIFF files resulting from the image acquisition into an IMS file.
32. Open file with the Imaris software (version 10.1 or above).
33. Explore the lymph node in 2D and 3D by clicking through the channels (Figure 5).

***Note:*** The quality of clearing should be ascertained. In Figure 5A, the signal intensity and contrast are constant across the Z axis of the sample, demonstrating that the clearing procedure was efficient. In contrast, a poorly cleared sample may show diffuse, low-contrast signal in deeper regions across all channels, regardless of focus adjustment. These areas may appear hazy or shadowed, and a dense, dark core may be visible where the light sheet cannot penetrate.

***Note:*** This protocol suggests using 5 markers which are useful for characterizing the germinal center reaction in immunized lymph nodes but, we have also optimized other markers which enable the characterization of stromal features like lymphatics, vasculature and the sinuses. Other optimized antibodies with example 3D and 2D images can be found in Figure 19. Troubleshooting 5.

***Note:*** Antibody specificity was confirmed using the appropriate controls, and no positive signal in the expected areas was detected. For conjugated antibodies (Bcl6-AF488, CD3-AF594, CD138-AF647, and IgD-AF700), fluorochrome-conjugated isotype controls were used (see Materials and Equipment). For CD21/35-AF790, a control lymph node was stained with the secondary antibody only (see Figure S1).

**Figure 6:**
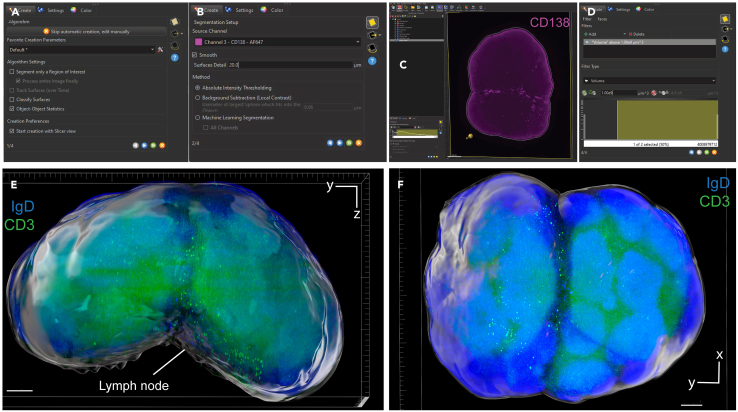
Surface creation of whole lymph node in Imaris (A) First step of the surface creation wizard: general algorithm settings (B) Second step of the surface creation wizard: source channel and method (C) Third step of the surface creation wizard: absolute intensity threshold (D) Fourth step of the surface creation wizard: filtering based on surface volume. (E and F) Representative 3D volume renderings of the lymph node surface using maximum intensity projection with a reference frame for orientation. The surface is rendered with transparency, allowing visualization of the underlying fluorescence channels: IgD (blue) and CD3 (green). In panel E, the bottom axis represents the y-axis, while the side axis represents the z-axis. In panel F, the bottom axis represents the y-axis, and the side axis represents the x-axis. Scale bar = 200 μm.

**Figure 7:**
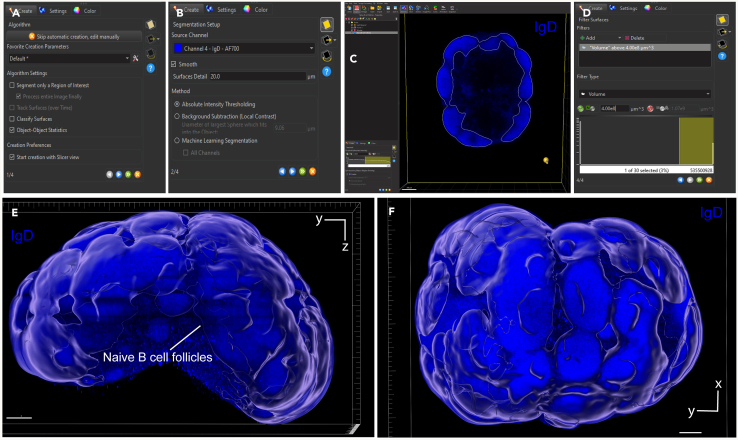
Naïve B cell surface creation using IgD fluorescence channel (A) First step of the surface creation wizard: general algorithm settings (B) Second step of the surface creation wizard: source channel and method (C) Third step of the surface creation wizard: absolute intensity threshold (D) Fourth step of the surface creation wizard: filtering based on surface volume. (E and F) Representative 3D volume renderings of the naïve B cell surface using maximum intensity projection with a reference frame for orientation. The surface is rendered with transparency, allowing visualization of the underlying fluorescence channel: IgD (blue). In panel E, the bottom axis represents the y-axis, while the side axis represents the z-axis. In panel F, the bottom axis represents the y-axis, and the side axis represents the x-axis. Scale bar = 200 μm.

**Figure 8:**
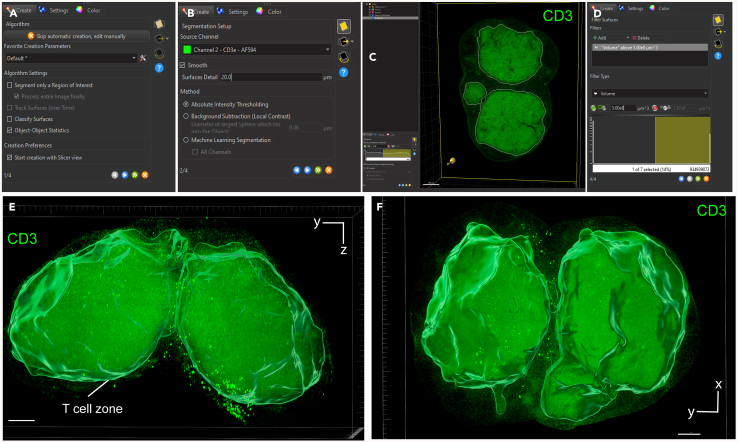
T cell zone surface creation using CD3 fluorescence channel (A) First step of the surface creation wizard: general algorithm settings (B) Second step of the surface creation wizard: source channel and method (C) Third step of the surface creation wizard: absolute intensity threshold (D) Fourth step of the surface creation wizard: filtering based on surface volume. (E and F) Representative 3D volume renderings of the T cell zone surface using maximum intensity projection with a reference frame for orientation. The surface is rendered with transparency, allowing visualization of the underlying fluorescence channel: CD3 (green). In panel E, the bottom axis represents the y-axis, while the side axis represents the z-axis. In panel F, the bottom axis represents the y-axis, and the side axis represents the x-axis. Scale bar = 200 μm.

**Figure 9:**
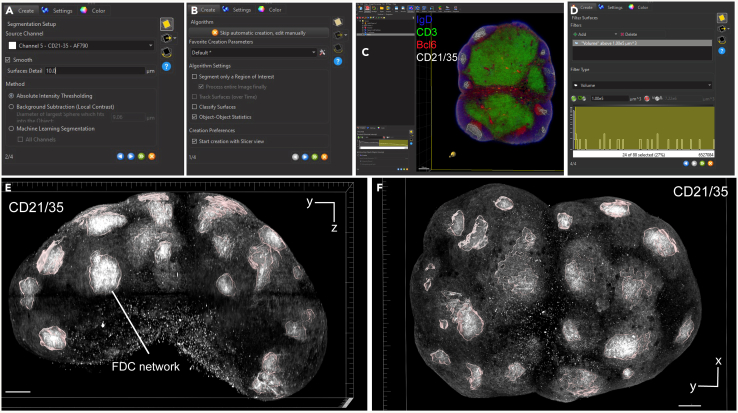
Segmentation of follicular dendritic cell networks using CD21/35 signal (A) First step of the surface creation wizard: general algorithm settings (B) Second step of the surface creation wizard: source channel and method (C) Third step of the surface creation wizard: absolute intensity threshold (D) Fourth step of the surface creation wizard: filtering based on surface volume. (E and F) Representative 3D volume renderings of the follicular dendritic cell network surface using maximum intensity projection with a reference frame for orientation. The surface is rendered with transparency, allowing visualization of the underlying fluorescence channel: CD21/35 (white). In panel E, the bottom axis represents the y-axis, while the side axis represents the z-axis. In panel F, the bottom axis represents the y-axis, and the side axis represents the x-axis. Scale bar = 200 μm.

**Figure 10:**
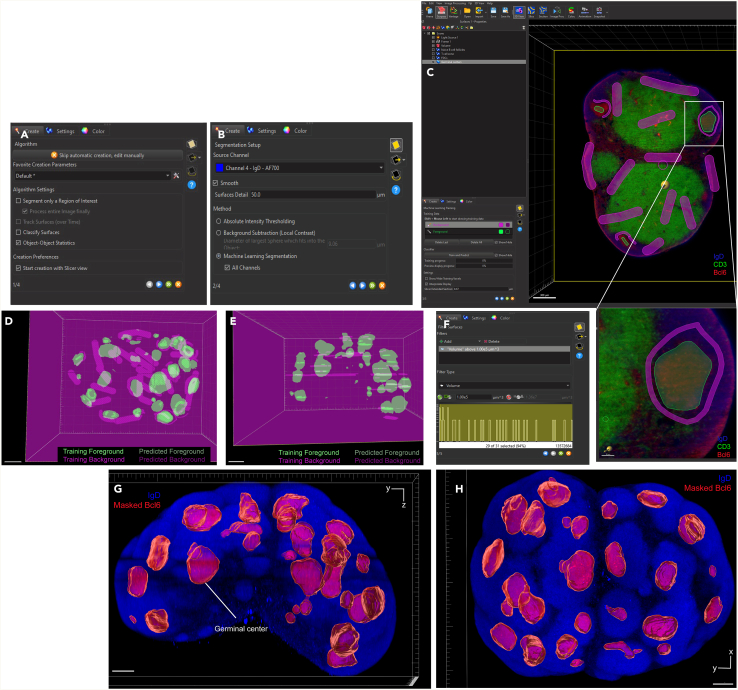
Machine learning-based segmentation of germinal centers and Bcl6 signal masking (A) First step of the surface creation wizard: general algorithm settings (B) Second step of the surface creation wizard: source channel and method (C) Third step of the surface creation wizard: Machine learning training by painting foreground and background brush strokes including inset showing a germinal center which has been cropped. (D and E) 3D renderings of training and predictive brush strokes. The lighter areas are training brush strokes, and the darker areas are the predicted classification. The pink color represents background, and the green color represents foreground. Scale bar = 300 μm. (F) Final step of the surface creation wizard: filtering based on surface volume. (G and H) Representative 3D volume renderings of the germinal centers surfaces and surface-masked Bcl6 channel (red) using maximum intensity projection with a reference frame for orientation. The surface is rendered with transparency, allowing visualization of the underlying fluorescence channels: IgD (blue) and masked Bcl6 (red). In panel G, the bottom axis represents the y-axis, while the side axis represents the z-axis. In panel H, the bottom axis represents the y-axis, and the side axis represents the x-axis. Scale bar = 200 μm.

**Figure 11:**
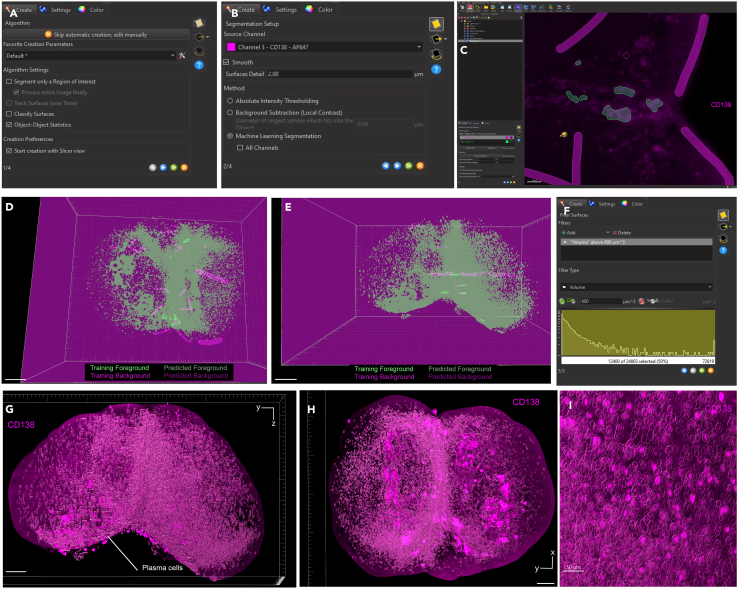
Plasma cell identification via CD138 channel using machine learning surface segmentation (A) First step of the surface creation wizard: general algorithm settings (B) Second step of the surface creation wizard: source channel and method. (C) Third step of the surface creation wizard: Machine learning training by painting foreground and background brush strokes. (D and E) 3D images of training and predictive brush strokes. The lighter areas are training brush strokes, and the darker areas are the predicted classification. The pink color represents background, and the green color represents foreground. (F) Final step of the surface creation wizard: filtering based on surface volume. (G and H) Representative 3D volume renderings of the plasma cell surfaces using maximum intensity projection with a reference frame for orientation. In panel G, the bottom axis represents the y-axis, while the side axis represents the z-axis. In panel H, the bottom axis represents the y-axis, and the side axis represents the x-axis. Scale bar = 200 μm. (I) Zoomed in 3D rendering of the plasma cell surfaces using maximum intensity projection. The surface is rendered with transparency, allowing visualization of the underlying fluorescence channel: CD138 (magenta). Scale bar = 50 μm.

**Figure 12:**
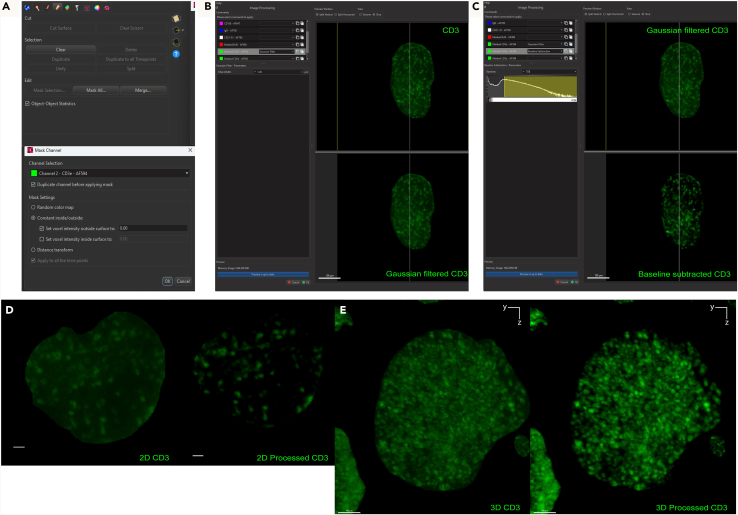
Preprocessing of CD3 channel to enable segmentation of germinal center–resident T cells (A) The germinal center surface is used to mask the CD3 channel by setting the voxel intensity of CD3 outside the germinal center surface to 0. (B) Gaussian Filter = 1 is applied to the masked CD3 channel. (C) Baseline subtraction = 750 is applied to the Gaussian filtered masked CD3 channel. Representative 2D planes (D) and 3D renderings using maximum intensity projection (E) of the masked CD3 channel in one germinal center before and after image processing. Scale bar in D = 20 μm. Scale bar in E = 100 μm.

**Figure 13:**
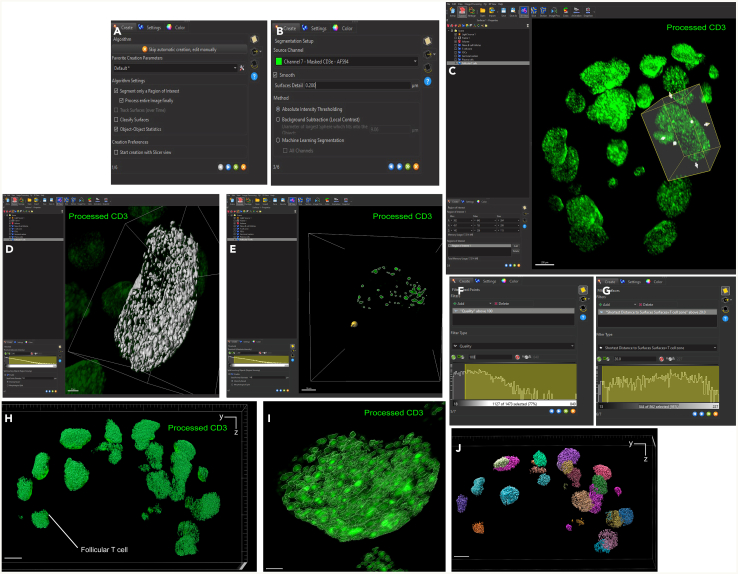
Segmentation and classification of follicular T cells within individual germinal centers (A) First step of the surface creation wizard: general algorithm settings (B) Second step of the surface creation wizard: source channel and method (C) Third step of the surface creation wizard: region of interest selection (D and E) Fourth step of the surface creation wizard: absolute intensity threshold. (F) Fifth step of the surface creation wizard: seed point filtering based on quality. (G) Final step of the surface creation wizard: surface filtering based on distance from T cell zone (H and I) Representative 3D volume renderings of the follicular T cells surfaces and surface-masked CD3 channel (green) using maximum intensity projection in the whole lymph node (H) and one cropped germinal center (I) with a reference frame for orientation. The surface is rendered with transparency, allowing visualization of the underlying fluorescence channel. In panel H, the bottom axis represents the y-axis, while the side axis represents the z-axis. Scale bar in H = 200 μm. Scale bar in I = 30 μm. (J) 3D representation of the follicular T cell surfaces, colored by Germinal Center ID. Scale bar = 200 μm.

34. Create the lymph node surface (Figure 6).
  a. Use the ‘Surface Object Creation’ module in Imaris. Process the entire image without choosing a region of interest, do not classify surfaces and enable object-object statistics (Figure 6A).
  b. Choose the channel with the most background signal (here CD138-AF647) as the source channel (Channel 3), enable Smooth (20 μm grain size) with Absolute Intensity Thresholding (Figure 6B).
  c. Choose a threshold which enables the segmentation of the whole lymph node. The inguinal lymph node has two lobes and is shaped like a kidney bean (Figure 6C).
  d. Filter surfaces by Volume (Figure 6D).
  e. Execute all creation steps (Figures 6E and 6F).
35. Create the Naïve B cell surface (Figure 7).
  a. Use the ‘Surface Object Creation’ module in Imaris. Process the entire image without choosing a region of interest, do not classify surfaces and enable object-object statistics (Figure 7A).
  b. Choose IgD-AF700 as the source channel (Channel 4), enable Smooth (20 μm grain size) with Absolute Intensity Thresholding (Figure 7B).
  c. Choose a threshold which enables the segmentation of only the naïve B cells. These should form a ring of oblong structures on the outside of the lymph node. Do not split surfaces (Figure 7C).
  d. Filter surfaces by Volume (Figure 7D).
  e. Execute all creation steps (Figures 7E and 7F).
36. Create the T cell zone surface (Figure 8).
  a. Use the ‘Surface Object Creation’ module in Imaris. Process the entire image without choosing a region of interest, do not classify surfaces and enable object-object statistics (Figure 8A).
  b. Choose CD3-AF594 as the source channel (Channel 2), enable Smooth (20μm grain size) with Absolute Intensity Thresholding (Figure 8B).
  c. Choose a threshold which enables the segmentation of only the T cell zone. The T cell zone should form two teardrops joined in the middle. Do not split surfaces (Figure 8C).
  d. Filter surfaces by Volume (Figure 8D).
  e. Execute all creation steps (Figures 8E and 8F).
37. Create the FDC network surfaces (Figure 9).
  a. Use the ‘Surface Object Creation’ module in Imaris. Process the entire image without choosing a region of interest, do not classify surfaces and enable object-object statistics (Figure 9A).
  b. Choose CD21-35-AF790 as the source channel (Channel 5), enable Smooth (10 μm grain size) with Absolute Intensity Thresholding (Figure 9B).
  c. Choose a threshold which enables the segmentation of only the FDC networks. These should coincide with the germinal centers visible by the Bcl6-AF488 and IgD-AF700 channels. Do not split surfaces (Figure 9C).
  d. Filter surfaces by Volume (Figure 9D).
  e. Execute all creation steps (Figures 9E and 9F).
38. Create the Germinal Center surfaces (Figure 10).
  a. Use the ‘Surface Object Creation’ module in Imaris. Process the entire image without choosing a region of interest, do not classify surfaces and enable object-object statistics (Figure 10A).
  b. Choose IgD-AF700 as the source channel (Channel 4), enable Smooth (50 μm grain size) with Machine Learning Segmentation with All channels (Figure 10B).
    ***Note:*** Because the AF488 channel exhibits the highest level of autofluorescence, it tends to show increased background staining compared to other channels. In particular, though most of the red blood cells are washed away during sample preparation, there is usually some vessel autofluorescence. For this reason, Bcl6 is visible within the germinal center but, it preferable to use the absence of IgD for the segmentation. It is possible to place Bcl6 on another less autofluorescent channel like AF647 for example (see Figure 16).
  c. Use the paintbrush tools to draw training data. Place green brush strokes in germinal centers and pink brush strokes outside of germinal centers (Figure 10C). Germinal centers, Bcl6^+^, are found between the IgD^+^ follicles, and the CD3^+^ T cell zone.
    **CRITICAL:** Be as precise as possible when creating the training data so the algorithm can correctly segment the germinal centers.
    ***Note:*** By saving the surface creation parameters, including the training data, the algorithm for segmentation can be stored, refined and reused for subsequent lymph nodes.
  d. Use training brushstrokes to predict. Continue the process iteratively until the result is satisfactory through the entire stack (Figures 10D and 10E).
  e. Do not split touching objects.
  f. Filter Surfaces by Volume to avoid artefacts (Figure 10F).
  g. Execute all creation steps (Figure 10G).
  h. For better visualization, use the Germinal Centers surface to mask the Bcl6-AF488 channel by duplicating the channel and setting the voxel intensity outside the surface to 0.00. This removes all the background Bcl6 autofluorescence. This will be Channel 6 (Figures 10H and 10I).
39. Create the Plasma cells surfaces (Figure 11).
  a. Use the ‘Surface Object Creation’ module in Imaris. Process the entire image without choosing a region of interest, do not classify surfaces and enable object-object statistics (Figure 11A)
  b. Choose CD138-AF647 as the source channel (Channel 3), enable Smooth (2 μm grain size) with Machine Learning Segmentation. Do not tick ‘All channels’ (Figure 11B).
  c. Use the paintbrush tools to draw training data. Place green brush strokes on plasma cells and pink brush strokes outside plasma cells (particularly on the outside ring of the lymph node which tends to show background fluorescence) (Figure 11C).
    ***Note:*** Machine learning segmentation is necessary here because plasma cells are found in the lymph node as either individual cells distributed throughout the T cell zone and near germinal centers or clustered in the sinus space. Clustered plasma cells have increased CD138 staining which makes it impossible to segment plasma cells based on intensity alone.
  d. Use training brushstrokes to predict. Continue the process iteratively until the result is satisfactory through the entire stack (Figures 11D and 11E).
  e. Do not split touching objects.
  f. Filter Surfaces by Volume (Figure 11F)
  g. Channel 3 mean intensity to avoid artefacts.
  h. Execute all creation steps (Figures 11G–11I).
40. Create the Follicular T cells surfaces (Figures 12 and 13).
  a. Use the Germinal Centers surface to mask the CD3 channel by duplicating the channel and setting the voxel intensity outside the surface to 0.00. This will be Channel 7 (Figure 12A).
  b. In the ‘Image Processing’ tab of Imaris, set the Gaussian Filter on this new channel to 1 (Figure 12B) and set the Baseline Subtraction using the histogram to remove non-specific background (Figure 12C).
    ***Note:*** The segmentation of T cells within the germinal center necessitates some image processing since single cells should be distinguished. First, mask the CD3-AF594 channel to only show the CD3-AF594 signal inside the germinal centers. Since CD3 is a cytoplasmic marker, we use Gaussian filter and Baseline subtraction to facilitate segmentation (Figures 12D and 12E).
  c. Use the ‘Surface Object Creation’ module in Imaris. Choose a region of interest, do not classify surfaces and enable object-object statistics (Figure 13A).
    ***Note:*** If desired, it is possible to classify follicular T cells based on their overlapped volume ratio with the FDC surface (these would be light zone follicular T cells). In this case, enable classification of surfaces.
  d. Choose Channel 7 as the source channel, enable Smooth (0.2 μm grain size) with Absolute Intensity Thresholding Set the region of interest to one germinal center with representative CD3 staining (Figure 13B).
  e. Set the region of interest to one germinal center with representative CD3 staining (Figure 13C).
  f. Choose a threshold which enables the segmentation of only the follicular T cells. Enable intensity-based surface splitting with a seed points diameter of 7 μm (Figures 13D and 13E).
  g. Filter seed points by quality. Most seed points will be correct (Figure 13F).
  h. Filter surfaces by Shortest Distance to Surfaces = T cell zone to exclude T cell zone T cells from segmentation (Figure 13G).
  i. Execute all creation steps.
  j. Follicular T cells can be associated to the germinal center they belong to by using the classification tool with the circle selection mode. Assign all the follicular T cells in one germinal center to a class named with that germinal center’s ID (Figure 13J).
    ***Note:*** To store the surface creation parameters to apply them to another image acquired in the same batch, navigate to the wand icon under the surface of interest and click “Store Parameters for Batch”. This can be done for each of the surfaces segmented here and enables consistency across images acquired together. This step also incorporates all of the training data for any surfaces segmented using machine learning.
    ***Note:*** Throughout creation steps, it is useful to toggle between 3D and 2D visualizations.

### Image analysis: Statistics export

**Timing: 5–10 min per lymph node**

**Figure 14:**
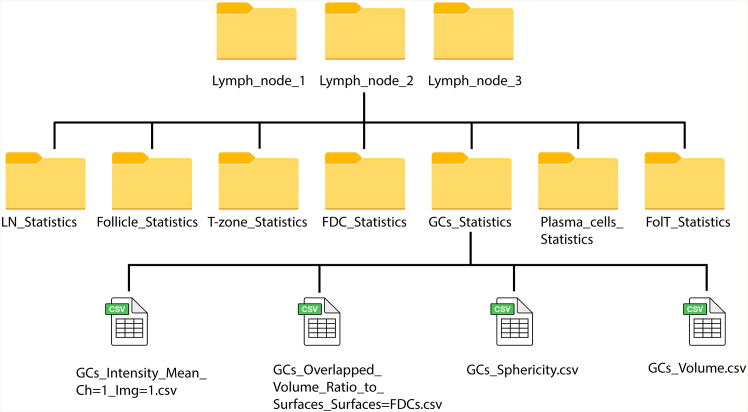
Schematic of folder hierarchy for exporting statistics from Imaris The above hierarchy must be followed to ensure compatibility with the provided R workflow.

Here, we describe the use of our custom R workflow housed on the Imaris-3D-LN-stats GitHub repository to compile statistics from the same surface across different images, including the necessary file organization structure (Figure 14).

41. Export statistics from all surfaces.
  **CRITICAL:** To ensure compatibility with the custom R workflow provided, files should be organized in a structure similar to Figure 14.
  a. For each surface, click the statistics tab.
    ***Note:*** Use the settings to select which statistics to export.
  b. Click Export all Statistics to File.
  c. Save the file using the surface name as the file name (e.g. GCs) and save within a folder named uniquely for that image (e.g. Lymph_node_1). Each statistic (e.g., ‘Volume’, ‘Mean Channel Intensity’, ‘Overlapped Volume Ratio to Surfaces’) will be in a separate csv file within the statistics folder. (Figure 14)
    **CRITICAL:** Ensure you export the same set of statistics across different images for a given surface using the same file name (e.g. GCs).
42. Use custom R workflow in command line to compile statistics for the same surface across different images. See the GitHub repository for detailed documentation including an example (see key resources table).
  a. Prepare the input CSV file with the following two columns: Sample_ID which is the image name (e.g. Lymph_node_1) and Directory which is the path to the directory which contains the statistics you would like to compile (e.g. .test/Lymph_node_1/GCs_Statistics).
  b. In command line, clone the repository from the associated GitHub page by running the following code.
    > git clone https://github.com/FrancisCrickInstitute/Imaris- 3D-LN-stat
    > cd ./Imaris-3D-LN-stats
  c. Compile the csv files, by executing the compiler script. The following arguments should be specified: <input_csv_path> is the path to the input CSV file, <output_csv_path> is the destination path for the compiled output CSV and <column_to_merge1> is the classification column name (optional).
    > /path/to/Rscript compiler.R <input_csv_path> <output_csv_path> <column_to_merge1 (optional)> <column_to_merge2 (optional)>
    ***Note:*** If you have classified objects in Imaris, specify the classification column name(s) so the compiler script can group statistics by Object ID and Classification.
  d. The resulting csv file will be exported to the specified directory and will include for each Object all of the exported statistics as well as a unique identifier column.

## Expected outcomes

**Figure 15:**
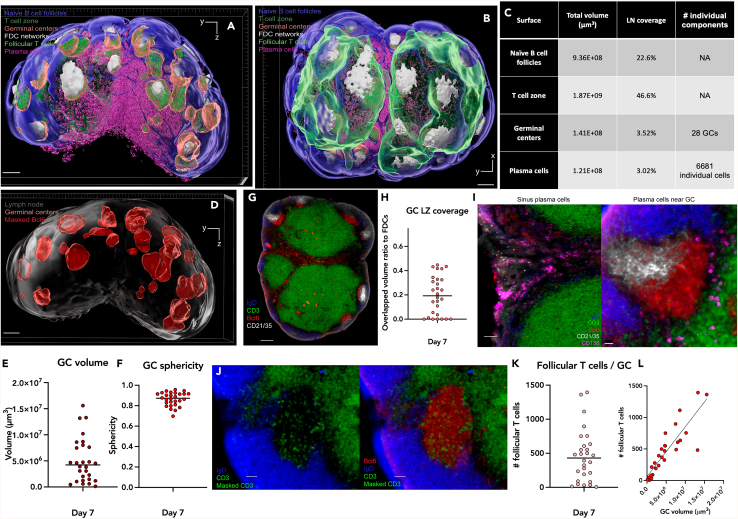
Quantitative 3D analysis of germinal centers, plasma cells, and follicular T cells post immunization (A and B) Representative image of 3D rendered surfaces created with Imaris including naïve B cells (blue), the T cell zone (green), follicular dendritic cell (FDC) network (white), germinal centers (red), follicular T cells (green) and plasma cells (magenta). In panel A, the bottom axis represents the y-axis, while the side axis represents the z-axis. In panel B, the bottom axis represents the y-axis, and the side axis represents the x-axis. Scale bar = 200 μm. (C) Table showing exported statistics from the rendered surfaces. Individual plasma cells were quantified by counting the number of Plasma cell surfaces which measured less than 3000 μ^3^ in volume. (D) Representative 3D image showing the lymph node and germinal center surfaces. The germinal center surface is rendered with transparency, allowing visualization of the underlying fluorescence channel: Germinal center masked Bcl6 (red). Scale bar = 200 μm. (E) Volume (μ^3^) of individual germinal centers (F) Sphericity of individual germinal centers. (G) Representative 2D image of lymph node slide showing germinal centers (Bcl6: red) with variable FDC (CD21/35: white) coverage. Scale bar = 100 μm. (H) Overlapped volume ratio of germinal centers to FDC network surface. (I) Representative cropped 2D images of areas with positive CD138 (magenta) staining. Plasma cells can be seen clustered in the sinuses between the T cell zone (CD3: green) or near germinal centers (Bcl6: red). 1^st^ panel scale bar = 70 μm, 2^nd^ panel scale bar = 40 μm. (J) Representative 2D images with or without Bcl6 (red) which shows individual follicular T cells (Masked CD3: green) within one germinal center. (K) Number of follicular T cells per germinal center. Scale bar = 40 μm. (L) A dot plot illustrating the relationship between germinal center volume (x-axis) and the number of follicular T cells (y-axis), with a fitted line representing a simple linear regression.

This optimized protocol enables the imaging and analysis of whole immunized lymph node germinal centers and associated populations. Figure 15 shows an example of one lymph node 7 days post-immunization. The segmentation procedure enables 3D rendering of the key immune populations and structures in the germinal center reaction (Figures 15A and 15B).

The segregation of the lymph node into B cell follicles and a distinct T cell zone is visible. Most of the B cell follicles contain one germinal center, characterized by the absence of IgD and the expression of Bcl6. The relative lymph node coverage of the naïve B cell follicles, the T cell zone, the germinal centers and the plasma cells is shown (Figure 15C).

Germinal center (Figure 15D) morphometric measurements like volume (Figure 15E) and sphericity (Figure 15F) can be assessed. Imaris segmentation identified 28 germinal centers which matched the number counted manually by the author. The FDC networks cover approximately 25% of the surface of the germinal centers, demarcating the light zone (Figures 15G and 15H).

Plasma cells are visible both clustered in the sinuses and in the vicinity of germinal centers (Figure 15I). This protocol allows the visualization of individual plasma cells, meaning it achieves single-cell resolution. This is possible because cells are not too densely packed. The total volume occupied by plasma cells and the number of individual plasma cells can be quantified by counting the number of plasma cell surfaces under 3000 μm^3^ (Figure 14C).

Masking of the CD3 channel with the germinal center surface and downstream image processing enables the segmentation of individual follicular T cells which are then classified by the germinal center they belong to (Figure 15J). The number of follicular T cells for each germinal center is shown (Figure 15K). As expected, germinal center volume is significantly positively correlated to the number of follicular T cells (Pearson’s correlation test, R^2^ = 0.76, p < 0.0001, Figure 15L). In the sample shown in the figure, the total number of follicular T cells amounted to 12,859 which is within the range one would expect based on flow cytometric quantification of PD1^+^CXCR5^+^ CD4^+^ T cells of lymph nodes post-immunization (approximately 10,000 cells).^13^

## Quantification and statistical analysis

Statistics from Imaris are exported into separate folders for each segmented surface, with individual CSV files containing different statistical measures. This structure makes data analysis cumbersome, as it requires extensive manipulation. To streamline this process, we provide an R workflow (R version 3.6+) that rapidly consolidates all Imaris statistics from a single segmented surface from the same image. We also provide the code to combine the statistics from the same surface across different images into a unified data frame, including a unique identifier column for each image. Detailed documentation can be found in the associated GitHub repository (see key resources table).

This workflow requires the installation of the general R program. However, all steps can be executed entirely via the command line. Users can then conduct statistical analyses directly in R or export the data frame as a CSV file for further analysis in GraphPad Prism or similar software. This approach is particularly beneficial for analyzing segmented surfaces such as Germinal Centers, Follicular T Cells, and Plasma Cells, where each image contains numerous distinct objects for each surface.

## Limitations

**Figure 16:**
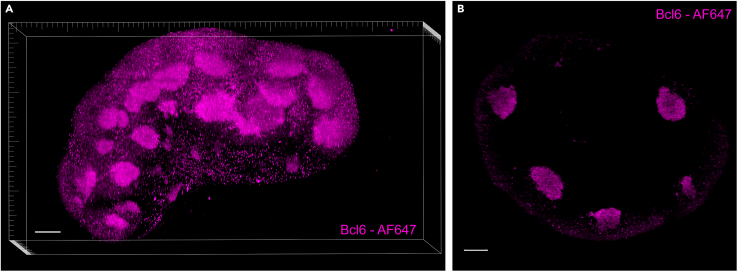
Reduced autofluorescence in lymph nodes using Bcl6-AF647 conjugate for improved germinal center detection Example 3D volume rendering (A) and 2D plane (B) of an inguinal lymph node stained with anti-mouse Bcl6 conjugated to AF647. (A) A frame is provided for orientation. The bottom axis represents the y-axis, while the side axis represents the z-axis. Scale bar = 200 μm.

While this protocol enables the imaging of lymph node germinal center reactions across five fluorescence channels, certain limitations should be considered. The AF488 channel, in particular, is highly susceptible to autofluorescence, which can result in background signal, particularly from vasculature due to autofluorescent red blood cells. As this protocol focuses on segmenting germinal centers which can be done using the IgD channel, rather than all Bcl6^+^ cells, this issue is not critical for its intended application. However, the AF488 channel should be avoided for markers with unknown expression patterns to prevent misinterpretation. Alternatively, perfusing the animal with PBS before harvesting the tissues has been shown to reduce autofluorescence by clearing the vasculature of red blood cells. Moreover, it is possible to use an anti-mouse Bcl6 antibody conjugated to another fluorophore such as AF647 if CD138 is not required (Figure 16).

Additionally, while optimized for lymph node staining, clearing, and imaging, this protocol may not be directly applicable to other lymphoid tissues such as the spleen or bone marrow. The spleen’s extensive vascularization and the bone marrow’s lower permeability to clearing agents could impact imaging outcomes and require further optimization.

One of the key strengths of this protocol is its ability to characterize five distinct intracellular and extracellular markers in whole mount lymph nodes without relying on genetic labeling tools. While other protocols and primary research articles have imaged similar lymph node structures and cell populations (such as germinal centers, follicular T cells, and plasma cells) using light sheet fluorescence microscopy, they have typically done so by using genetically modified mice where specific populations are fluorescently labeled (e.g., AID.Cre.tdTomato for germinal center B cells, Blimp1-YFP for plasma cells) or by adoptively transferring immune cells from genetically modified mice where all cells are fluorescently labeled (GFP^+^ or CFP^+^ mice).^14^^,^^15^ While these approaches are valuable in some contexts, this protocol allows for the investigation of lymph node populations regardless of genetic composition. This makes it particularly useful for studies involving diverse mouse genotypes (e.g., cancer or autoimmunity murine models) and removes the need for adoptive transfers or complex breeding strategies.

However, having said this, it has not been optimized to conserve endogenous fluorescent signal. Indeed, methanol and BABB clearing can reduce native fluorophore signals. Alternative clearing approaches, such as dehydration with tert-butanol at pH 9.5 (instead of methanol) and titration of BABB to pH 9.5 with triethylamine, have been reported to preserve endogenous fluorescence.^16^ However, titrating BABB is not straight-forward and adapting this protocol in this way may require multiple rounds of optimization. On the other hand, immunolabeling with antibodies against endogenous fluorescent proteins (e.g., anti-GFP or anti-mCherry) may provide an alternative means of detecting labeled cells.

While this protocol enables the visualization and quantification of single plasma cells and follicular T cells in the germinal center, this is largely due to the relatively low cell density in these regions. Therefore, while the protocol achieves single-cell resolution for sparsely distributed cells, its applicability may be limited for densely packed populations, such as T cells in the T cell zone or naïve B cells in the B cell follicles. In cases where single-cell resolution of densely packed populations is required, the use of a confocal microscope may be more appropriate than a light sheet fluorescence microscope. However, it is worth noting that imaging a single whole lymph node with a confocal microscope can take up to 12 hours, whereas the same sample can be imaged in approximately 30 minutes using a light sheet fluorescence microscope. This makes acquiring in batches very challenging and thus, voxel intensity across samples cannot be easily compared. An alternative approach may be to increase the zoom setting on the light sheet fluorescence microscope to its maximum and image only a selected portion of the lymph node.

It is worth noting that methanol dehydration and BABB clearing leads to tissue shrinkage. Thus, it is recommended to monitor the sample size and match the timing of these steps carefully across experiments to enable the comparison of volumetric and distance measurements. This avoids differential tissue shrinkage between sample batches.

Finally, users should be aware that the Imaris software used for analysis can be prone to crashes, potentially leading to data loss. It is strongly recommended to save work frequently, especially when creating new channels or surfaces. This issue may be mitigated by using a computer with a high-performance CPU (3.3 GHz with 12 to 16 cores), ample RAM (64-128 GB), a dedicated GPU with sufficient VRAM. Additionally, using an SSD for storage can improve stability and performance. While Imaris is proprietary software, open-access tools like FIJI, StarDist, CellPose, and napari are advancing the field of image analysis and improving accessibility. However, these alternatives are still in development and may not yet match the full capabilities of commercial solutions.

## Troubleshooting

### Problem 1: Distorted or torn tissue

Tissue distortion or tearing may result from overfixation or improper handling. Overfixation can compromise tissue integrity, so reducing fixation time may help mitigate this issue. Additionally, while lymph nodes may appear intact externally, excessive handling can cause internal structural damage (relevant to “Mouse immunization, lymph node harvest and fixation” and “Permeabilization, staining, embedding and clearing”).

### Potential solution

To prevent this, samples should be handled as minimally as possible after cleaning, and direct compression or squeezing should be avoided.

### Problem 2: Antibody aggregates

**Figure 17:**
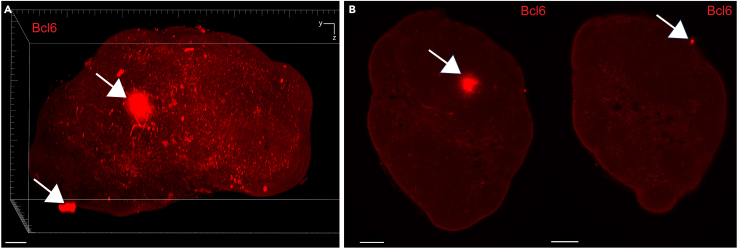
Detection of antibody aggregates as sources of nonspecific background in Bcl6 channel Example 3D volume rendering (A) and 2D planes (B) of a naïve lymph node showing antibody aggregation. The channel shown is Bcl6-AF488 (red). (A) A frame is provided for orientation. The bottom axis represents the y-axis, while the side axis represents the z-axis. Scale bar = 150 μm. (B) Scale bar = 200 μm.

Antibody aggregation within the tissue can lead to areas of intense background fluorescence that obscure signal detection. These aggregates appear as highly bright regions in images (Figure 17) (relevant to “Permeabilization, staining, embedding and clearing” and “Light sheet microscope acquisition”).

### Potential solution

To reduce this issue, the antibody cocktail should be centrifuged before use to pellet any aggregates. Further improvements can be achieved by increasing blocking times, adding additional washing steps, or reducing antibody concentrations.

### Problem 3: Weak signal or lack of staining

A weak signal or absence of staining can arise from various factors. Bcl6-AF488 staining, in particular, may appear dim relative to background autofluorescence in the same channel (relevant to “Permeabilization, staining, embedding and clearing” and “Light sheet microscope acquisition”).

### Potential solution

To enhance signal intensity, the anti-mouse Bcl6 antibody can be included in both Antibody cocktail 1 and Antibody cocktail 2, thus increasing the staining duration and improving detection. Alternatively, the antibody concentration can be increased.

### Problem 4: Photobleaching

**Figure 18:**
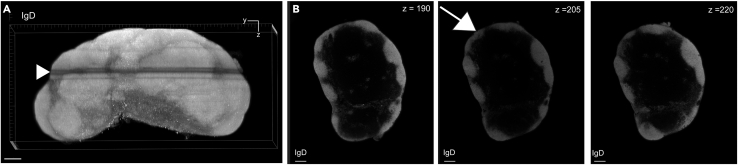
Photobleaching artifacts in IgD channel during light sheet imaging Example 3D volume rendering (A) and 2D planes (B) of a lymph node showing photobleaching. The channel shown is IgD-AF700 (blue) and the photobleached z stacks can be seen as a black band through the tissue (A) which look darker when observed in 2D with the same brightness settings (B). In (B), the panel marked with an arrow highlights one of the photobleached z stacks where the signal intensity is reduced compared to the other panels. (A) A frame is provided for orientation. The bottom axis represents the y-axis, while the side axis represents the z-axis. Scale bar = 200 μm.

High laser intensity is sometimes necessary to achieve enough signal-to-noise ratio but can lead to photobleaching, particularly in channels such as IgD-AF700 where the fluorophores used are less photostable. Photobleaching often appears as a dark band within the 3D rendering using maximum intensity projection (Figure 18) (relevant to “Light sheet microscope acquisition”).

### Potential solution

To minimize this effect, adjustment of the focus on the sample must be done using a low laser power and by gently moving the sample across the light-sheet plane rather than remaining on a single z-plane for an extended period. For IgD-AF700 imaging, setting the focus at the top or bottom of the z-stack rather than within the middle of the sample may also help preserve fluorescence signal.

### Problem 5: Insufficient marker coverage

The five markers outlined in this protocol may not fully capture the structures or cell populations of interest (relevant to “Permeabilization, staining, embedding and clearing” and “Image analysis: Surface segmentation”).

### Potential solution

**Figure 19:**
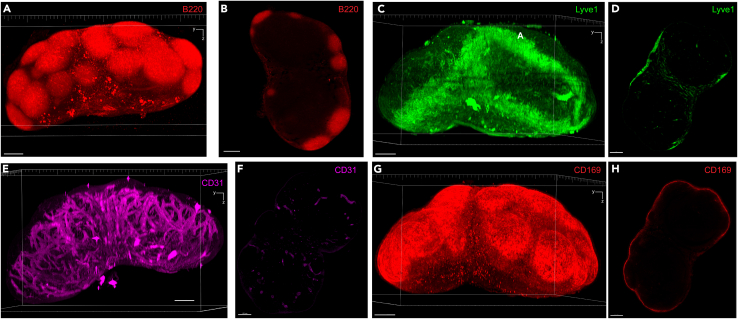
Visualization of additional stromal and immune cell populations using optimized antibodies Example 3D volume renderings and 2D planes. Anti-mouse B220-AF594 enables visualization of all B cells (A and B). Anti-mouse Lyve1-AF488 captures the lymphatics (C and D). Anti-mouse CD31-AF647 enables visualization of the lymph node vasculature (E and F). Anti-mouse CD169-AF594 captures subcapsular sinus macrophages (G and H). In the 3D volume renderings using maximum intensity projection, a frame is provided for orientation. The bottom axis represents the y-axis, while the side axis represents the z-axis. Scale bar in all images = 200 μm.

We have optimized additional marker stainings (Figure 19).These include markers for all B cells (B220, Figures 19A and 19B), lymphatics (Lyve1, Figures 19C and 19D), vasculature (CD31, Figures 19E and 19F) and the subcapsular sinus (CD169, Figures 19G and 19H). Additionally, while specific fluorophore-marker combinations are recommended in this protocol, antibodies of the same clone conjugated to different fluorophores can be substituted as needed.

### Problem 6: Selecting markers for 3D imaging

**Figure 20:**
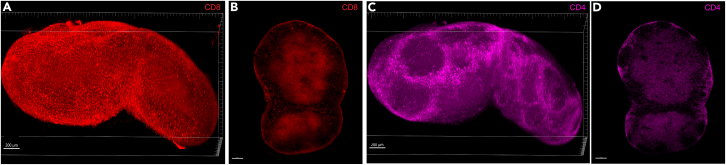
Limitations of CD4 and CD8 markers for defining T cell zones in 3D imaging Example 3D volume renderings (A and C) and 2D planes (B and D) of CD8 (A and B) and CD4 (C and D) in mouse inguinal lymph nodes. While the T cell zone is visible in 2D, it is obscured in 3D by cell populations which also express CD4 and CD8 but, are most likely not T cells. In the 3D volume renderings using maximum intensity projection, a frame is provided for orientation. The bottom axis represents the y-axis, while the side axis represents the z-axis. Scale bar = 200 μm.

When selecting markers for 3D immunofluorescence staining, it is important to consider that markers effective in other contexts may not perform as well in 3D imaging. For example, CD4 and CD8 are commonly used to identify T cell subsets in flow cytometry (with an additional CD3 antibody) and in 2D immunofluorescence, where other cell types expressing these markers can typically be ignored. However, in 3D renderings, non-T cells that also express CD4 or CD8 and can be located on the outside of the lymph node, can obscure the true T cell zone, making it harder to interpret the data. For example, conventional dendritic cells (cDC1) express CD8^17^ and subcapsular sinus macrophages can express CD4.^18^ This issue is illustrated in Figure 20 (relevant to “Permeabilization, staining, embedding and clearing” and “Image analysis: Surface segmentation”).

### Potential solution

Before incorporating a new marker into a 3D staining panel, review its known expression profile and test it in 2D immunofluorescence to assess specificity. Markers which are uniquely expressed by the cell type of interest are preferable. When introducing new antibodies, include appropriate staining controls such as fluorescently labeled isotype controls or secondary antibody-only controls.

## Resource availability

### Lead contact

Further information or requests for resources and reagents should be directed to and will be fulfilled by the lead contact, Dinis Calado (dinis.calado@crick.ac.uk).

### Technical contact

For protocol details or additional inquiries, email the technical contact, Victoire Boulat (victoire.boulat@crick.ac.uk)

### Materials availability

This study did not generate any new unique reagents.

### Data and code availability

Requests for the datasets generated in the optimization of this protocol should be directed to and will be fulfilled by the lead contact, Dinis Calado (dinis.calado@crick.ac.uk). The custom code generated to consolidate statistics exported from Imaris is available from the Imaris-3D-LN-stats_2025 repository on the Francis Crick Institute GitHub (https://github.com/FrancisCrickInstitute/Imaris-3D-LN-stats, https://doi.org/10.5281/zenodo.16789543).

## Acknowledgments

Work in the laboratory was supported by the FCI, which receives core funding from Cancer Research UK (grants CC2078 and CC1069), the UK Medical Research Council (grants CC2078 and CC1069), and the Wellcome Trust (grants CC2078 and CC1069) to D.P.C. and the Advanced Light Microscopy facility); the UK Biotechnology and Biological Sciences Research Council (grant BB/W016427/1 to D.P.C.); and the UK Medical Research Council (grant MR/W025221/1 to D.P.C. and A.G.). V.B. was supported by the CRUK City of London Centre Award CANTAC721\100023. Drawings were created using BioRender.com.

## Author contributions

V.B. conceived the protocol, with support from A.C., M.S.H., and L.Z. A.G. and D.P.C. acquired funding and supervised the study. All authors participated in the writing of the manuscript.

## Declaration of interests

The authors declare no competing interests.
