# Supplementary material for: Protocol for rapid 5-plex 3D imaging and single-cell analysis of immune responses in whole murine lymph nodes
Source: STAR Protoc. 2025 Sep 1;6(3):104059. doi: 10.1016/j.xpro.2025.104059 (PMC12423407; doi:10.1016/j.xpro.2025.104059)
Supplement: Document S1. Figure S1 [file mmc1.pdf]

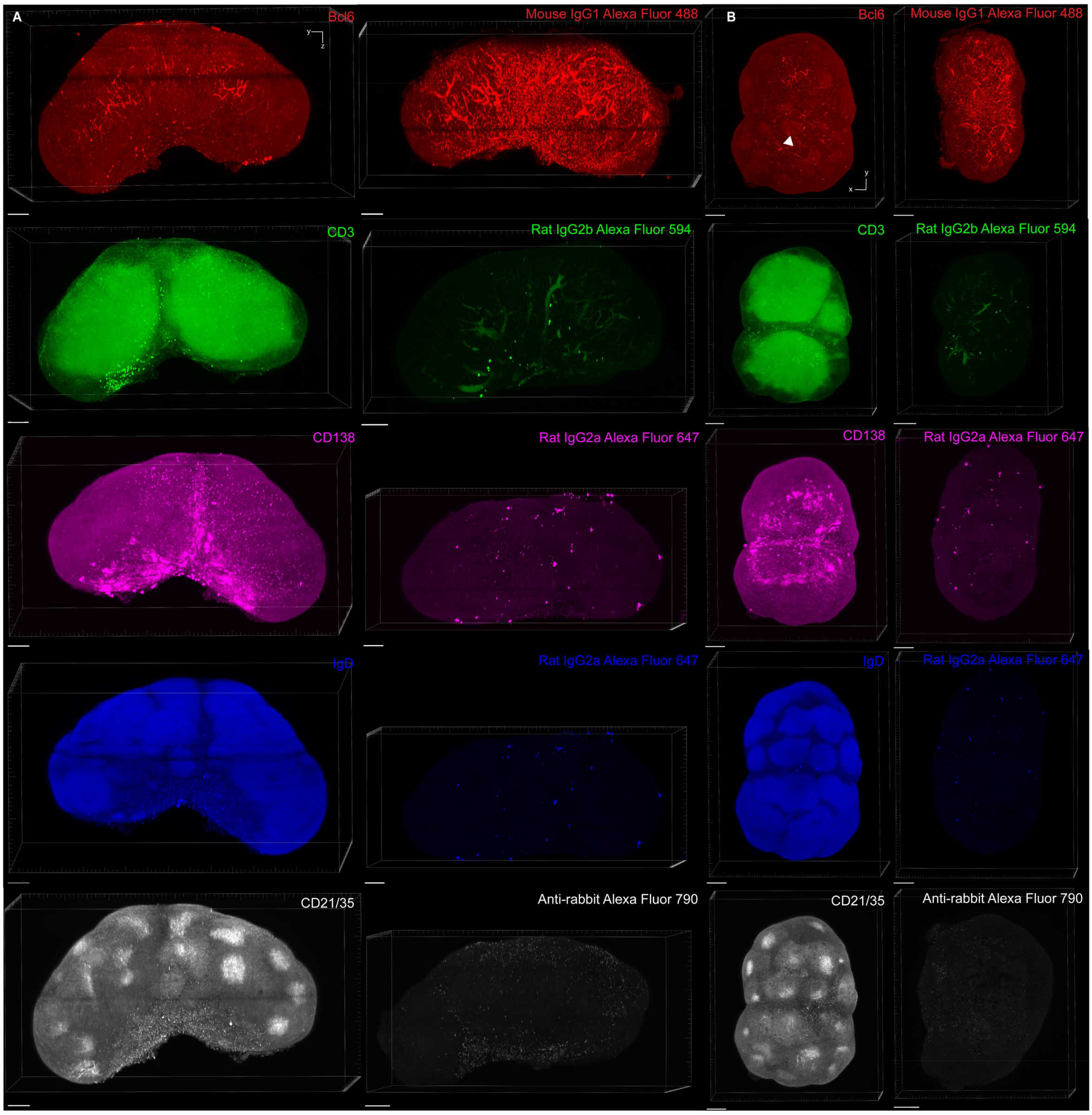

Supplemental Figure 1. Assessment of Staining Specificity in 3D Lymph Node Imaging with Isotype and Secondary-Only Controls, related to Figure 5. All images are 3D renderings shown as maximum intensity projections. In the first two columns (A), the bottom axis represents the y-axis, while the side axis represents the z-axis. In the last two columns (B), the bottom axis represents the x-axis, and the side axis represents the y-axis. The laser brightnesses used to acquire the control lymph node images matched those used for the fully stained lymph node. The display settings are matched within channels to enable comparison, and the display brightness minimum is set to 0 to include background signal. As discussed elsewhere, the channel with the highest background signal is the Bcl6-Alexa Fluor 488 channel, due to autofluorescence of vessels and cells (see Figure 15 for an alternative). This is not a major issue since the IgD Alexa Fluor 700 channel is used for the detection of germinal centers. Despite this, in all channels, there is no positive signal in the control lymph nodes within the expected structures or cell types, confirming that the positive signal in the stained lymph nodes within the expected areas is real. A scalebar = 200 μm, B scalebar = 300 μm.
